# Inference about causation from examination of familial confounding (ICE FALCON): a model for assessing causation analogous to Mendelian randomization

**DOI:** 10.1093/ije/dyaa065

**Published:** 2020-06-04

**Authors:** Shuai Li, Minh Bui, John L Hopper

**Affiliations:** 1 Centre for Epidemiology and Biostatistics, Melbourne School of Population and Global Health, University of Melbourne, Melbourne, VIC, Australia; 2 Centre for Cancer Genetic Epidemiology, Department of Public Health and Primary Care, University of Cambridge, Cambridge, UK

**Keywords:** Mendelian randomization, causal inference, ICE FALCON, twin study, instrumental variable

## Abstract

**Background:**

We developed a method to make Inference about Causation from Examination of FAmiliaL CONfounding (ICE FALCON) using observational data for related individuals and considering changes in a pair of regression coefficients. ICE FALCON has some similarities to Mendelian randomization (MR) but uses in effect all the familial determinants of the exposure, not just those captured by measured genetic variants, and does not require genetic data nor make strong assumptions. ICE FALCON can assess tracking of a measure over time, an issue often difficult to assess using MR due to lack of a valid instrumental variable.

**Methods:**

We describe ICE FALCON and present two empirical applications with simulations.

**Results:**

We found evidence consistent with body mass index (BMI) having a causal effect on DNA methylation at the *ABCG1* locus, the same conclusion as from MR analyses but providing about 2.5 times more information per subject. We found evidence that tracking of BMI is consistent with longitudinal causation, as well as familial confounding. The simulations supported the validity of ICE FALCON.

**Conclusions:**

There are conceptual similarities between ICE FALCON and MR, but empirically they are giving similar conclusions with possibly more information per subject from ICE FALCON. ICE FALCON can be applied to circumstances in which MR cannot be applied, such as when there is no a priori genetic knowledge and/or data available to create a valid instrumental variable, or when the assumptions underlying MR analysis are suspect. ICE FALCON could provide insights into causality for a wide range of public health questions.

**Key Messages**

Inference about Causation from Examination of FAmiliaL CONfounding (ICE FALCON) uses observational data for related individuals, in particular twin pairs, to assess causality between measured variables.
ICE FALCON has some similarities to Mendelian randomization (MR) but uses in effect all the familial determinants of the exposure, not just those captured by measured genetic variants, and does not make strong assumptions. ICE FALCON and MR empirically are giving similar conclusions with possibly more information per subject from ICE FALCON.
ICE FALCON does not require genetic data and can be applied to circumstances in which MR cannot be applied, such as when there is no a priori genetic knowledge and/or data available to create a valid instrumental variable for the exposure of interest, or when the assumptions underlying MR analysis are suspect.

## Background

Mendelian randomization (MR) uses measured genetic variants as instrumental variables for exposures to make inference about causation from observational data. The methodology behind MR has developed substantially over the past decade,^1^^,^^2^ and the increasing application of MR is made possible by genome-wide association studies (GWAS) and the greater open access of GWAS data to the research community. It is notable that the instrumental variables used in MR are familial, in that they are correlated between blood relatives. This raises the prospect of thinking about MR in the context of family designs, including twin pairs.

Twin studies have special properties for understanding the causes of variation of traits. Traditionally, twin studies have been used to test hypotheses about unmeasured causes. For example, comparing the similarity of monozygotic (MZ) twin pairs with the similarity of dizygotic twin pairs for a particular trait is a way of testing the null hypothesis that genetic factors do not influence variation in that trait. Under certain assumptions, these twin studies can also be used to estimate the proportion of variation due to genetic and shared non-genetic factors.

The within-twin pair designs, using pairs discordant for outcome or exposure^3^ and the within-pair differences in continuous traits,^4^^,^^5^ have been used to test causal hypotheses about measured causes. The value of these designs is that they control for potential confounders, including familial confounders. Given this strength, it is argued that an observed association is more likely to be causal. However, some possibilities cannot be ruled out, such as reverse causation when using cross-sectional data, and unmeasured confounding even when using longitudinal data.

We have developed a new method, Inference about Causation from Examination FAmiliaL CONfounding (ICE FALCON), which applies to data for related individuals and enables an assessment of evidence for causality and causal direction between measured factors—using statistical analysis to try to detect the signal from the noise. ICE FALCON has been applied to try to understand the causes of several traits including mammographic density,^6^^,^^7^ allergic conditions,^8^ psychological behaviours,^9^ bone architecture^10^ and epigenetic modifications.^11^^,^^12^ In the latter papers we compared our findings with those from MR, and found they agreed. ICE FALCON has similarities and differences to MR; see Discussion.

In this paper we describe the methodology of ICE FALCON. To illustrate how it can be applied, we present simulation studies and two examples.

For the first example, we considered the cross-sectional association between body mass index (BMI) and blood DNA methylation level at site *cg06500161* of the *ABCG1* locus. MR analyses have suggested that this association is due to a causal effect of BMI on methylation: Wahl *et al.*^13^ reported that a BMI polygenic risk score (PRS) based on 29 variants was associated with methylation level (*P *=* *6.4 × 10^–5^), by analysing data for 4034 individuals; Mendelson *et al.*^14^ analysed data for 2170 individuals and found that a BMI PRS based on 97 variants was associated with methylation level (*P *=* *7.1 × 10^–3^).

For the second example, we considered the issue of causality between BMI measures repeated in time. This addresses whether a correlation between BMI at two different time points is due to: (i) BMI at the earlier time having a causal effect on BMI at the later time; (ii) familial factors (genetic and/or non-genetic) that operate at both time points; or (iii) non-familial factors that operate at both time points. For MR to assess these explanations of tracking with time, there would need to be genetic variants associated with BMI at the earlier time which are not associated with BMI at the later time. Genetic variants for adulthood BMI found to date by GWAS appear to apply to BMI across the whole of adulthood;^15^ there are as yet no validated genetic variants associated with BMI at an earlier age which are not associated with BMI at a later age. Even genetic variants found to be associated with childhood BMI are associated with adulthood BMI.^16^

## Methods

### ICE FALCON

Suppose there are two traits, *X* and *Y*, measured for related individuals. Here we assume, for simplicity of exposition and the examples, that the related individuals are twin pairs but other pairs of related individuals, such as sibling pairs, could also be used. We consider here only one exposure variable, but the extension to multiple exposures is straightforward.

**Figure 1:**
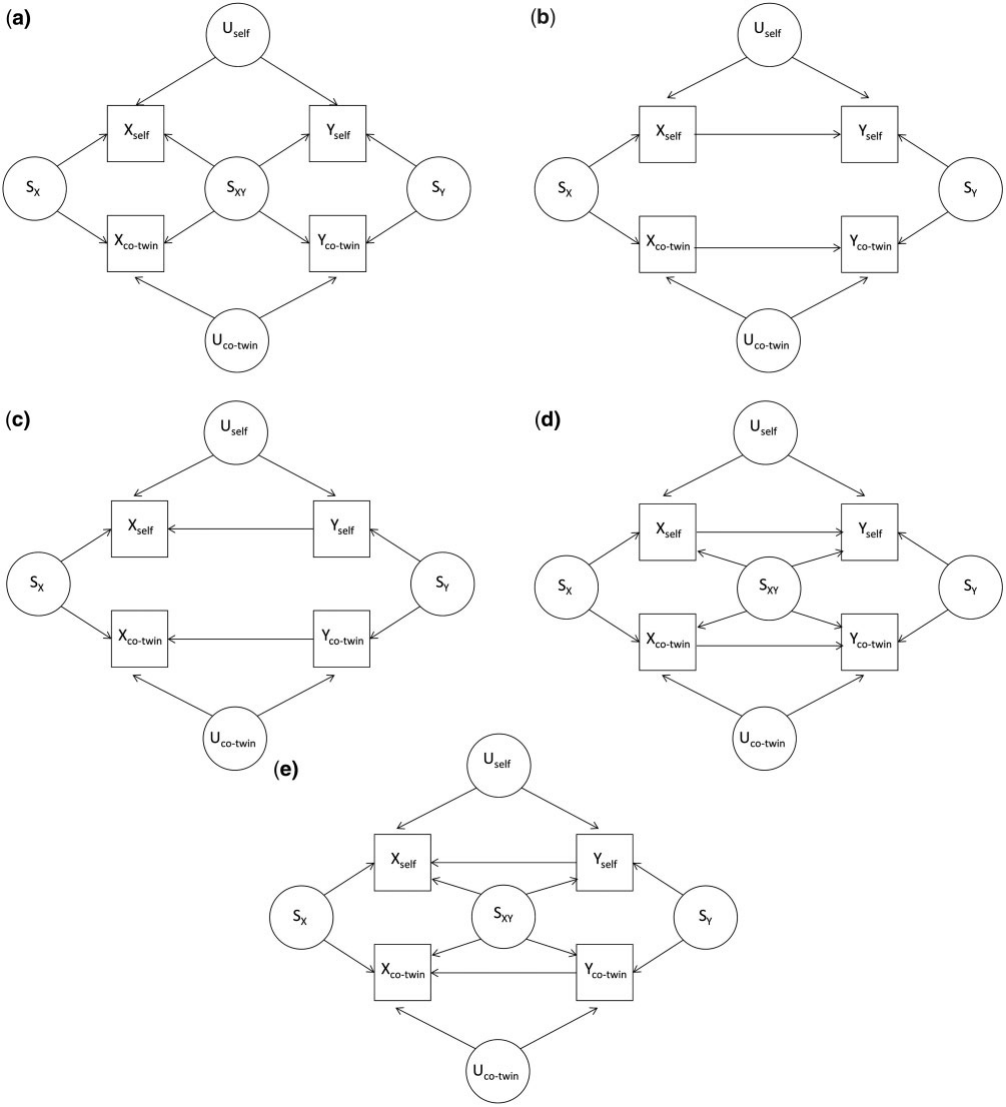
Possible causal diagrams for two measured traits *X* and *Y* for twins.

Without loss of generality, assume that *X* and *Y* are positively associated within an individual. Figure 1 shows some possible causal diagrams for *X* and *Y*, in which circles are unmeasured causes and squares are measured traits following the original convention.^17^ These diagrams combine the original path analysis concept introduced by Sewall Wright to study genetic and environmental causes of variation^17^ with the directed acyclic graphs used in current epidemiology.^18^ Let *S* denote all the combination of unmeasured causes (genetic and/or non-genetic) that affect both twins; *S_X_* represents those causes that influence *X* only, *S_Y_* those causes that influence *Y* only and *S_XY_* those causes (familial confounders) that influence both *X* and *Y*. Let *U* denote all the unmeasured individual-specific confounders between *X* and *Y* which are not shared by twins. For the purposes of explanation, let ‘self’ refer to an individual and ‘co-twin’ refer to the individual’s twin, but recognize that these labels can be swapped and both twins within a pair are included in the analysis.

If a cross-twin cross-trait correlation (the correlation between *Y_self_* and *X_co-twin_*, or between *X_self_* and *Y_co-twin_*) exists, it might be due to the following: (i) the effects of familial confounders *S_XY_* (Figure 1a); (ii) causation between *X* and *Y*, provided *X_self_* and *X_co-twin_* are correlated due to *S_X_* and/or *Y_self_* and *Y_co-twin_* are correlated due to *S_Y_* (Figure 1b and c); or (iii) a mixture of familial confounding and causation between *X* and *Y* (Figure 1d, e).

The trait *X* is assumed to be the predictor variable and *Y* is assumed to be the outcome variable (the roles of *X* and *Y* can be reversed in order to provide additional evidence; see below). Three models are fitted:

$$\text{Model 1}:E(Y_{self})=\alpha +\beta_{self}X_{self}$$

 

$$\text{Model 2}:E(Y_{self})=\alpha +\beta_{co-twin}X_{co-twin}$$

 

$$\text{Model 3}:E(Y_{self})=\alpha +{\beta^{\prime }}_{self}X_{self}+{\beta^{\prime }}_{co-twin}X_{co-twin}$$

For the purpose of illustration, the models are simplified without including any covariates, though covariates can be easily included. Given that both twins within a pair are included in the regression, the correlation in the outcome variable between twins needs to be accounted for. This can be handled by explicitly estimating the correlation, using for example the package FISHER,^19^^,^^20^ or by applying a random-effects model or a generalized estimating equations (GEE) model.

If there is familial confounding only (i.e. no direct causation) (Figure 1a), there will be associations between *Y_self_* and *X_self_* (*β_self_*, Model 1), and between *Y_self_* and *X_co-twin_* (*β_co-twin_*, Model 2). Adjusting for *X_self_* (Model 3), there will still be a conditional association between *Y_self_* and *X_co-twin_* (*β'_co-twin_*), but it will be attenuated towards the null compared with *β_co-twin_*. Similarly, adjusting for *X_co-twin_* (Model 3), the conditional association between *Y_self_* and *X_self_* (*β'_self_*) will be attenuated towards the null compared with *β_self_*. Both the attenuations will be of a similar magnitude.

If there is a causal effect from *X* to *Y* only (Figure 1b), there will be an association between *Y_self_* and *X_self_* (*β_self_*, Model 1). In Model 2, *Y_self_* and *X_co-twin_* are associated through two paths: the confounder *S_X_*, and conditioning on the collider *Y_co-twin_* (accounting for the correlation in *Y* between twins in effect conditions on *Y_co-twin_*). Conditioning on *Y_co-twin_* induces a negative correlation between *X_co-twin_* and *Y_self_*, so that *β_co-twin_* depends on the within-pair correlations in *X* (*ρ_X_*) and in *Y* (*ρ_Y_*): if *ρ_X_*>*ρ_Y_*, *β_co-twin_* is expected to be positive; otherwise to be negative. Conditioning on *X_self_* (Model 3), both paths are closed and the conditional association between *Y_self_* and *X_co-twin_* (*β'_co-twin_*) will be null, and therefore attenuated compared with *β_co-twin_*. However, conditioning on *X_co-twin_* (Model 3), the association between *Y_self_* and *X_self_* (*β'_self_*) will be similar to *β_self_*.

If there is a causal effect from *Y* to *X* only (Figure 1c), there will be an association between *Y_self_* and *X_self_* (*β_self_*). In Model 2, there is no open path between *Y_self_* and *X_co-twin_*—the path through *X_self_* is closed due to *X_self_* being a collider, and the path through *S_Y_* is closed due to the fact that *Y_co-twin_* is conditioned on, so *β_co-twin_* is null. Conditioning on *X_self_* (Model 3), both paths are open and there will be a conditional association between *Y_self_* and *X_co-twin_* (*β'_co-twin_*), which depends on *ρ_X_* and *ρ_Y_*: if *ρ_X_*>*ρ_Y_*, *β'_co-twin_* is expected to be negative; otherwise to be positive.

The mathematical formulae for the theoretical arguments above can be found in the Supplementary data, available at *IJE* online.

If there is both familial confounding and causation (Figures 1d and e), the results will be a mixture of the results mentioned above. The changes in the pair of regression coefficients from comparing Model 3 with Models 1 and 2 will apply, allowing assessment of evidence for causality still to be made. Of course, the possibility that these changes are consistent with chance needs to be assessed by applying formal statistical inference, and this can be achieved using bootstrapping (see Statistical Methods) or simulation studies.

In summary, for the different causal diagrams showed by Figure 1, distinct patterns of changes in regression coefficients are expected (Table 1). Therefore, formal hypothesis testing of the changes in the pair of regression coefficients can be used to assess the strength, and the statistical significance, of the evidence for one or more causal diagrams being consistent with the data.

**Table 1.** The expected results from ICE FALCON analysis of regressing Y on X for different causal scenarios

Model	Coefficient	Familial confounding	X causing Y	Y causing X
Model 1	β_self_	Association	Association	Association
Model 2	β_co-twin_	Association	Association	No association
Model 3	β’_self_	Association; attenuated towards the null compared with β_self_ of Model 1	Association; the same as β_self_ of Model 1	Association
	β’_co-twin_	Association; attenuated towards the null compared with β_co-twin_ of Model 2, to the same extent as the attenuation of β’_self_ compared with β_self_	No association; attenuated to the null compared with β_co-twin_ of Model 2	Association; negative if ρ_X_ >ρ_Y,_ otherwise positive

To provide additional evidence, *X* and *Y* can be reversed, i.e. let *Y* be the predictor variable and *X* be the outcome variable and fit the same three regression models. For Figure 1a, the same results as those when using *X* as the predictor variable are expected. For Figure 1b, the results are expected to be the same as those for Figure 1c when using *X* as the predictor variable; similarly, the results for Figure 1c are expected to the same as those for Figure 1b when using *X* as the predictor variable. Therefore, if there is a causal association, the results from the analyses using *Y* as the predictor variable are expected to differ in distinct ways from those from the analyses using *X* as the predictor variable.

Note that, ICE FALCON does not model the within-pair correlations in *X* or *Y*, as in a classic twin model that assumes no casual effect between *X* and *Y*. ICE FALCON investigates the changes in regression coefficients under different causal scenarios, and thereby has the potential to uncover novel information about the source of the data than could be found from fitting a model based only on correlations.

### Simulation studies

To test the validity of ICE FALCON in general, we simulated two causal scenarios: (i) *X* has a causal effect on *Y*; (ii) *Y* has a causal effect on *X*. For each scenario, we simulated *X* and *Y* to have various within-pair correlations, from 0.1 to 0.9 with a step of 0.1, and applied ICE FALCON. Details can be found in the Supplementary data, available at *IJE* online.

To show how the simulation studies can be used to help make inference about causation, for each of the two examples above we simulated three different causal scenarios, each based on the observed correlational structure of the data, and applied ICE FALCON. For Example 1, we simulated data consistent with BMI and DNA methylation level being associated due to: (i) familial confounding; (ii) BMI having a causal effect on DNA methylation level; and (iii) DNA methylation level having a causal effect on BMI. For Example 2, we simulated data being consistent with longitudinal BMI measures being associated due to: (i) familial confounding;(ii) the baseline measure having a causal effect on the follow-up measure; and (iii) a mixture of (i) and (ii), with weights of 36% and 64% as suggested by the emprical data analysis, respectively. We created a test statistic for each scenario based on the observed changes in regression coefficient estimates with those expected based on the simulations, taking into account the expected variation in estimates, and thereby derived empirical tests of fit with statistical significance. Null hypothesis of the test is that the simulated scenario is consistent with the observed results from empirical data analysis. Details can be found in the Supplementary data, available at *IJE* online.

### Subjects and materials

For the two examples, we used data from the Australian Mammographic Density Twins and Sisters Study, a twin and family cohort.^21^ Between 1995 and 1999 (baseline), female twins aged between 40 and 70 years were recruited and surveyed.^22^ Between 2004 and 2009 (follow-up), the twins were asked to participate again, and their sisters were also invited to participate. Participants at follow-up completed a survey and donated blood samples.^21^ Blood DNA methylation was measured using the Infinium HumanMethylation450 BeadChip array.^23^

For Example 1, we used follow-up data for 65 MZ pairs whose average age was 55.4 (standard deviation: 8.3) years, average BMI was 26.3 (5.4) kg/m^2^ and average methylation beta-value at *cg06500161* was 0.62 (0.03). We used the residuals in BMI after adjusting for age and smoking status, and residuals in methylation level after adjusting for age, smoking status and cell proportions in analysis. The within-pair correlations in BMI and in methylation level residuals were 0.79 [95% confidence interval (CI): 0.77, 0.82] and 0.37 (95% CI: 0.19, 0.53), respectively.

For Example 2, we used baseline and follow-up data for 250 MZ pairs whose average age was 49.6 (standard deviation: 7.4) years and average BMI was 24.8 (4.2) kg/m^2^ at baseline, and whose average age was 57.3 (7.3) years and average BMI was 25.6 (4.3) kg/m^2^ at follow-up. We used the residuals in the two BMI measures after adjusting for age in analysis.

### Statistical methods

The regression analyses were conducted using the GEE model with the exchangeable correlation structure. Standard error for the change in regression coefficient estimates between models was estimated using a nonparametric bootstrap method, in which the included twin pairs were randomly sampled with replacement to generate 1000 new datasets with the same sample size as the original dataset. ICE FALCON was then applied to each dataset to calculate the change in regression coefficient for that dataset. The standard error was the standard deviation of the change across the 1000 datasets.

## Results

### Simulation study

Under the scenario of *X* having a causal effect on *Y* (Supplementary Table S1, available as Supplementary data at *IJE* online), *X_self_* was associated with *Y_self_* in Model 1. *X_co-twin_* was associated with *Y_self_* in Model 2, and the estimate of *β_co-twin_* was positive when *ρ_X_* > *ρ_Y_*, and negative when *ρ_X_* < *ρ_Y_*. Conditioning on *X_self_* (Model 3), *X_co-twin_* was not associated with *Y_self_*, and the estimate of *β’_co-twin_* was close to null, regardless of *ρ_X_* or *ρ_Y_*. That is, there was a marginal association between *X_co-twin_* and *Y_self_*, and the association attenuated to the null after conditioning on *X_self_*.

Under the scenario of *Y* having a causal effect on *X* (Supplementary Table S2, available as Supplementary data at *IJE* online), *X_self_* was associated with *Y_self_* in Model 1. *X_co-twin_* was not associated with *Y_self_* in Model 2, and the estimate of *β_co-twin_* was close to the null, regardless of *ρ_X_* or *ρ_Y_*. Conditioning on *X_co-twin_* (Model 3), *X_co-twin_* was associated with *Y_self_*, and the estimate of *β’_co-twin_* was negative when *ρ_X_* > *ρ_Y_*, and positive when *ρ_X_* < *ρ_Y_*. That is, there was no marginal association between *X_co-twin_* and *Y_self_*, but there was an association after conditioning on *X_self_*.

Therefore, this simulation study showed that ICE FALCON gives distinct patterns of regression coefficients consistent with our theoretical arguments and the mathematical expressions.

### Example 1

Table 2 shows the results using BMI as the predictor variable and methylation level as the outcome variable. A woman’s methylation level was associated with her own BMI (Model 1; *β_self_* = 0.13, 95% CI: 0.05, 0.22) and with her co-twin’s BMI (Model 2; *β_co-twin_* = 0.09, 95% CI: 0.01, 0.17). Conditioning on her co-twin’s BMI (Model 3), the association between her methylation level and her own BMI remained unchanged (*P *=* *0.49). On the other hand, conditioning on her own BMI (Model 3), the association between her methylation level and her co-twin’s BMI attenuated by 97% to become null (*β'_co-twin_* = 0.003, 95% CI: −0.11, 0.12). This attenuation was marginally significant (*P *=* *0.08). The findings that there was an association between a woman’s methylation level and her co-twin’s BMI in Model 2, and that the association disappeared after conditioning on her own BMI, are consistent with the expectation of Figure 1b.

**Table 2.** Results from the ICE FALCON analysis for BMI and blood DNA methylation level at site *cg06500161* of the *ABCG1* locus

Predictor	Coefficient	Model 1		Model 2		Model 3		**Change** ^b^	
		Est (SE)	*P*	Est (SE)	*P*	Est (SE)	*P*	Est (SE)	*P*
BMI^a^	β_self_	0.13 (0.04)	2.1 × 10^−3^			0.13 (0.06)	0.03	−0.001 (0.06)	0.49
	β_co-twin_			0.09 (0.04)	4.9 × 10^−2^	0.003 (0.06)	0.96	−0.08 (0.06)	0.08
DNA methylation level	β_self_	26.6 (13.0)	4.8 × 10^−2^			39.8 (16.3)	0.02	14.2 (7.0)	0.02
	β_co-twin_			0.6 (12.1)	0.96	24.4 (14.6)	0.10	23.8 (7.9)	1.4 × 10^−3^

ICE FALCON, Inference about Causation through Examination of FAmiliaL CONfounding; BMI, body mass index; Est, estimate; SE, standard error.

a Regression results were reported as the change in percentage methylation per one unit increase in BMI.

b One-sided *P*-value.

We reversed BMI and methylation level, i.e. methylation level became the predictor variable and BMI was the outcome variable, and fitted the same three regression models (Table 2). A women’s BMI was associated with her own methylation level (Model 1; *β_self_* = 26.6, 95% CI: 0.1, 51.1), but not with her co-twin’s methylation level (Model 2; *β_co-twin_* = 0.6, 95% CI: −23.1, 24.3). However, after conditioning on her own methylation level, there was a marginally significant association between her own BMI and her co-twin’s methylation level (Model 3; *β'_co-twin_* = 24.4, 95% CI: −4.2, 53.0, *P *=* *0.1), and there was a significant change (*P *=* *1.4 × 10^–3^) when comparing *β'_co-twin_* with *β_co-twin_*. Given there was no association between a woman’s BMI and her co-twin’s methylation level in Model 2, but a change in association from conditioning on her own methylation level, these results are inconsistent with the expectation of Figure 1b, but consistent with the expectation of Figure 1c.

Therefore, the data are inconsistent with methylation level having a causal effect on BMI, but consistent with BMI having a causal effect on methylation level.

This inference is also supported by the simulation study; no evidence was found that the scenario in which BMI has a causal effect on DNA methylation level was inconsistent with the observed results (*P *=* *0.74), whereas the other two scenarios were inconsistent with the observed results (both *P *<0.05) (Supplementary Table S3 and Figure S1, available as Supplementary data at *IJE* online).

### Example 2

Table 3 shows that a woman’s follow-up BMI was associated with her own baseline BMI (Model 1; *β_self_* = 0.81, 95% CI: 0.72, 0.90), and with her co-twin’s baseline BMI (Model 2; *β_co-twin_* = 0.73, 95% CI: 0.65, 0.81). In Model 3, there remained a strong association between a woman’s follow-up BMI with her own baseline BMI (*β'_self_* = 0.73, 95% CI: 0.63, 0.83), and a weak association with her co-twin’s baseline BMI (*β'_co-twin_* = 0.15, 95% CI: 0.06, 0.23). Both the associations attenuated compared with the estimates from Models 1 and 2, but to different extents (*P *=* *1.8 × 10^–14^), being 9.8% (*P *=* *0.02) and 79.9% (*P *=* *1.5 × 10^–30^), respectively.

**Table 3.** Results from the ICE FALCON analysis for longitudinal BMI measures

Coefficient	Model 1		Model 2		Model 3		**Change** ^a^	
	Est (SE)	*P*	Est (SE)	*P*	Est (SE)	*P*	Est (SE)	*P*
β_self_	0.81 (0.04)	<2.0 × 10^−16^			0.73 (0.05)	<2.0 × 10^−16^	−0.08 (0.04)	0.02
β_co-twin_			0.73 (0.04)	<2.0 × 10^−16^	0.15 (0.04)	1.0 × 10^−3^	−0.59 (0.05)	<2.0 × 10^−16^

ICE FALCON, Inference about Causation through Examination of FAmiliaL CONfounding; BMI, body mass index; Est, estimate; SE, standard error.

a One-sided *P*-value.

The findings that there were associations for both a woman’s baseline BMI and her co-twin’s baseline BMI in Model 3, and that both the associations attenuated from comparing with Models 1 and 2 but to different extents, are consistent with the expectations of Figure 1d, i.e. a mixture of causation and familial confounding. We, therefore, interpret these results as being consistent with a longitudinal causation, as well as a small amount of familial confounding, underlying the association between the two longitudinal BMI measures.

This inference is also supported by the simulation study; no evidence was found that the scenario in which longitudinal BMI measures are associated due to a mixture of familial confounding and longitudinal causation was inconsistent with the observed results (*P *=* *0.35), whereas the other two scenarios were highly inconsistent with the observed results (both *P *=* *0) (Supplementary Table S4 and Figure S2, available as Supplementary data at *IJE* online).

## Discussion

We found from Example 1 that the ICE FALCON approach gave the same conclusion as from previous MR analyses, i.e. BMI has a causal effect on the blood DNA methylation level at the *ABCG1* locus. Our previous applications of ICE FALCON to data on exposures and blood DNA methylation also gave the same conclusion as from MR analyses.^11^^,^^12^

One measure of the amount of information on causality assessment from MR can be derived from consideration of the test statistic (*Z_MR_*) for the association between PRS and outcome, in proportion to the square root of the sample size (*n*). Similar for ICE FALCON, a measure of the amount of information comes from the test statistic (*Z_IF_*) for change in cross-trait cross-pair regression coefficient. The study by Wahl *et al.*^13^ had *n* = 4034 and *Z_MR_* = 4.00, the study by Mendelson *et al.*^14^ had *n* = 2170 and *Z_MR_* = 2.69 and our ICE FALCON analysis had *n *= 130 and *Z_IF_* = 1.75. Therefore, *Z_MR_/n*^1/2^ = 0.063 and 0.058 respectively when using MR, whereas *Z_IF_/n*^1/2^ = 0.153 when using ICE FALCON. That is, in this example ICE FALCON appears to be extracting about 2.5 times more information on causality per subject than MR. Given these *Z* scores capture the main driver of decision making for each method, we think *Z/n*^1/2^ is a good starting point for comparing the power per subject between the two methods, and this is an issue for further research.

We found from Example 2 that the longitudinal tracking in BMI is mostly consistent with a causal effect of BMI on its future values, as well as a smaller component of familial confounding. This implies that most of the reason why BMI is correlated in MZ pairs in later life is because they were correlated in earlier life. That is, the genetic and non-genetic factors relevant to BMI, which are shared by individuals in earlier life, have a lingering effect on BMI into their later life due to their BMI in earlier life having a causal effect on their future BMI. It also implies that BMI intervention studies can be effective, contrary to what would have been the implication had we found no evidence of longitudinal causation. Therefore, intervention studies on BMI may not necessarily be doomed to failure due to a deterministic interpretation of the effects of a person’s underlying genetic and other familial factors, as would be the conclusion had there been no evidence for longitudinal causation.

### Comparison of ICE FALCON and MR

ICE FALCON is analogous in some ways to MR. Consider the scenario in which *X* has a causal effect on *Y* (Figure 1b); *S_X_* is an instrumental variable for *X_self_*, the exposure, and includes all the familial determinants of *X*, not just the proportion captured by measured genetic variants. *S_X_* is not measured, but in this scenario a proxy measure is *X_co-twin_*. ICE FALCON studies the association between the proxy instrumental variable and the outcome, *Y_self_*. MR uses measured genetic variants within *Sx* as an instrumental variable. When the roles of *X* and *Y* are additionally reversed, ICE FALCON is analogous to a bidirectional MR analysis—the association between the proxy instrumental variable (*Y_co-twin_*) for outcome (*Y_self_*) and the exposure (*X_self_*) is also investigated.

Although sharing some similarities, a major differentiating point is that ICE FALCON does not rely on the strong assumptions of MR, and it makes inference based on changes in a pair of regression estimates, rather than estimation of a single parameter alone. MR makes the essential assumption that the measured genetic variants associated with *X* are all within *S_X_*, and that none are within *S_XY_*. ICE FALCON allows for *S_XY_* to exist. MR tests for causality by fitting a single parameter, whereas ICE FALCON considers a pair of parameters and how their estimates change between whether the parameters are estimated together or alone.

ICE FALCON, therefore, does not assume inference can be made based on a single ‘causal parameter’ related to a (genetic, and therefore familial) variable that is designated to be ‘instrumental’ by making strong assumptions which presume biological knowledge. Instead, ICE FALCON uses the existence of the familial similarity of an exposure to make causal inference using a new approach to hypothesis testing based on changes in pairs of regression coefficients. It is not even necessary to know the causes of the familial similarity of the exposure or decompose the expsoure's variance into genetic and/or non-genetic components, though the potential for obtaining new knowledge by doing so will be explored in future publications.

ICE FALCON is based on regression, so the method can be applied to continuous and binary outcomes using ordinary and logistic regression, respectively, and potentially to survival data using Cox regression. There are no restrictions on the measurement scale of exposures. ICE FALCON can also be used to assess the causes of tracking in a trait over time, as in Example 2, an issue that cannot be easily assessed using MR due to the difficulty of finding a valid instrumental variable.

The validity of an MR analysis is subject to three key assumptions.^1^  Table 4 summarizes a comparison between MR and ICE FALCON for each of these assumptions, and shows that ICE FALCON could have some advantages over MR.

**Table 4.** Comparison between MR and ICE FALCON with respect to the three key assumptions of MR

Assumptions (Ref.^1^)	MR	ICE FALCON
Relevance assumption: instrumental variable is strongly associated with the exposure	Measured genetic variants associated with exposure act as presumed instrumental variable Plausibility assessment: *F*-statistic, risk difference or using genetic variants found by large-scale GWAS to be associated with the exposure Weak instrumental variable bias	All unmeasured familial causes specific to the exposure act as instrumental variable; co-twin’s exposure variable used as a proxy Plausibility assessment: within-pair correlation in the exposure All familial causes specific to the exposure are stronger than a finite number of genetic variants associated with the exposure
Independence assumption: instrumental variable is independent of any confounder of the relationship between exposure and outcome	Validity assessment: biological knowledge, test between genetic variants and confounders, evidence from GWAS of confounders Approaches for invalidity: removing invalid genetic variants, adjusting for population stratification, adjusting for potential confounders	*X_co-twin_* is theoretically unrelated to unmeasured confounders specific to an individual only; any relation with unmeasured confounders shared between twins is captured by the familial confounder, *S_XY_* Still works even if *X_co-twin_* is also associated with *Y_self_* through *S_XY_*; inference on causation based on the changes in regression coefficients still apply
Exclusion restriction assumption: the association between the instrumental variable and the outcome is mediated through the exposure variable only	Validity assessment: biological knowledge, test between genetic variants and potential alternative mediators, evidence from GWAS of the outcome or potential alternative mediators Approaches for invalidity: removing invalid genetic variants, statistical methods such MR-Egger regression and Weighted Median Estimator	*X_co-twin_* is theoretically not related to *Y_self_* through potential alternative pathways in which *X_self_* is uninvolved Still works even if *X_co-twin_* is also associated with *Y_self_* through a mediator shared between twins; inference on causation based on the changes in regression coefficients still apply

Regarding the relevance assumption, MR requires the exposure to have been extensively studied, and measured, for genetic determinants. ICE FALCON, however, does not explicitly require genetic data and can be applied to all measured exposures of interest. There is a weak instrumental variable bias in MR estimates if the studied genetic variants do not explain a substantial variation in the exposure. By contrast, *S_X_* includes all causes of familial correlation in *X* that are specific to *X*, which is theoretically stronger than a limited number of genetic variants, so ICE FALCON will not be giving biased results in the way MR can.

Regarding the independence assumption, *X_co-twin_* is unrelated to *U_self_*, the individual-specific confounders of the relationship between *X_self_* and *Y_self_*. Any relationship with unmeasured confounders shared between twins is captured by *S_XY_*. Should *X_co-twin_* be related to *S_XY_*, as in the scenario showed by Figure 1d, ICE FALCON should still work — the association between *X_co-twin_* and *Y_self_* is still expected to attenuate towards the null after adjusting for *X_self_*, given that the path *X_co-twin_*←*S_X_* →*X_self_* →*Y_self_* is closed. Example 2 shows the validity of ICE FALCON in this scenario.

Similarly, regarding the exclusion restriction assumption, if *X_co-twin_* has directional pleiotropy, i.e. is related to *Y_self_* not through *X_self_* only but also through a shared mediator between twins, a change in the association between *X_co-twin_* and *Y_self_* is still expected after adjusting for *X_self_*.

There are, nonetheless, limitations in the usage of ICE FALCON. It requires data for related individuals with both members of the same pair having been measured in the same way for the variables of interest. ICE FALCON uses measured variables which can be subject to measurement error, whereas the genetic data used in MR typically have little measurement error.

Note that the measured exposure variables in ICE FALCON could also include measured genetic variants, so in principle ICE FALCON could be used to address the causality of polygenic risk scores and in theory even individual genetic variants. ICE FALCON and MR analyses could also be combined given a suitable dataset. The combination could be used to test the validity of some MR assumptions, such as whether the genetic variants for the exposure have directional pleiotropy. Methodology for the combination needs to be developed.

### Interpretation of results

As with MR analyses, results from ICE FALCON should of course be interpreted appropriately, given that they are both statistical modelling approaches which allow consideration of the extent to which the analysed data are consistent with different causal models. Neither approach can prove that a consistent model is a true representation of nature, and we are not proposing that ICE FALCON can ‘prove causality’. All that can be said is whether or not the data ‘are consistent with’ a particular causal hypothesis. The results of these observational analyses should be considered with other evidence as well when making interpretations, as pointed out by the guidelines (not criteria) developed by Bradford Hill for addressing causation based on assuming that a factor is causal and thinking through the consequences.^24^ ICE FALCON and MR are in effect doing the same thing, though more sophisticatedly than the usual approach of estimating associations from observational studies.

Nonetheless, statistical modelling is an attempt to identify the plausible and implausible explanations of data. ICE FALCON can be used to test hypotheses and thereby has the potential to falsify model(s). For example, classic bivariate twin models assume there are no causal effects between variables of interest. Attempts have been made to include causation,^25^ but those models do not consider causation and familial confounding together (i.e. they assume *S_XY_* does not exist). They also only use marginal correlations to make inference. Our analyses show that this assumption is not substantiated in either Example 1 or 2, because the observed regression coefficients clearly differ from those expected under the classical twin model. This calls into question the results of multivariate twin analyses that assume that the only reason why variables are correlated within pairs is due to shared familial factors, in effect excluding the potential for intervention studies.

### Further developments

Several issues need to be investigated to further develop ICE FALCON: (i) the statistical power of ICE FALCON; (ii) the change in regression coefficient in relation to the within-pair correlations in *X* and *Y*; (iii) quantifying the causal effect (ICE FALCON currently focuses on considering evidence for causality); and (iv) as mentioned above, how to combine ICE FALCON with MR.

To conclude: we have developed ICE FALCON, a statistical modelling approach to observational data for related individuals, to assess causality between measured variables of interest. There are some conceptual similarities and differences between ICE FALCON and MR, and empirically they are giving similar conclusions, with possibly more information per subject from ICE FALCON. ICE FALCON can be applied to circumstances in which MR cannot be applied, such as when there is no a priori genetic knowledge and/or data available to create a valid instrumental variable for the exposure of interest, or when the assumptions underlying MR analysis are suspect. ICE FALCON can also be used as an independent method to replicate the findings from MR analysis, and *vice versa*. By providing causality evidence in multiple ways, ICE FALCON, perhaps together with other causality assessing methods, should be useful in deciding, for example, whether to pursue intervention studies of the measured factor in relation to influencing the trait in question. Given ICE FALCON does not rely on genetic knowledge or measurement of those genetic factors, but instead uses the almost universal fact that siblings (and especially twins) are correlated in exposures, it could provide insights into causality for a wide range of public health questions.

## Funding

The Australian Mammographic Density Twins and Sisters Study (AMDTSS) was supported by the National Health and Medical Research Council (NHMRC, grant numbers 1050561 and 1079102), Cancer Australia and National Breast Cancer Foundation (grant number 509307). S.L. is a Victorian Cancer Agency Early Career Research Fellow (grant number ECRF19020). J.L.H. is a NHMRC Senior Principal Research Fellow. The work was supported by the NHMRC Program grant (grant number 1074383).

## Supplementary Material

dyaa065_Supplementary_Data
