# Supplementary material for: Inference about causation from examination of familial confounding (ICE FALCON): a model for assessing causation analogous to Mendelian randomization
Source: Int J Epidemiol. 2020 Jun 4;49(4):1259–69. doi: 10.1093/ije/dyaa065 (PMC7750993; doi:10.1093/ije/dyaa065)
Supplement: dyaa065_Supplementary_Data [file dyaa065_supplementary_data.pdf]

Mathematical expressions

We studied the three causal scenarios: 1) *X* and *Y* are associated due to familial confounding only (i.e. there is no direct causation), 2) *X* and *Y* are associated due to a causal effect of *X* on *Y* only, and 3) *X* and *Y* are associated due to a causal effect of *Y* on *X* only.

For a twin pair with twin 1 and twin 2, assume X_1 and Y_1 are the *X* and *Y* variables for twin 1, and X_2 and Y_2 are the *X* and *Y* variables for twin 2. For simplicity we assume that each X_1, X_2, Y_1 and Y_2 has zero mean and unit variance, there was no within-individual confounding between *X* and *Y*, the correlation between X_1 and X_2 is $\rho_{X_1X_2}$, the correlation between Y_1 and Y_2 is $\rho_{Y_1Y_2}$, the correlation between X_1 and Y_1 is $\rho_{X_1Y_2}$, and the correlation between X_1 and Y_2 is $\rho_{X_1Y_2}$. Note that, $\rho_{X_1Y_1} = \rho_{X_2Y_2}$, and $\rho_{X_1Y_2} = \rho_{X_2Y_1}$.

Under these assumptions, in all the three scenarios, from the Model 1,

$$\beta_{self} = \rho_{X_1Y_1} \tag{1}$$

1. X and Y are associated due to familial confounding only

From the Model 2,

$$\beta_{co-twin} = \rho_{X_2Y_1} \tag{2}$$

From the Model 3, based on partial correlation equation,

$$\begin{aligned}\beta'_{self} &= \rho_{X_1Y_1\,|X_2} \\ &= \frac{\rho_{X_1Y_1} - \rho_{X_1X_2}\,\rho_{Y_1X_2}}{\sqrt{1-\rho^2_{X_1X_2}}\sqrt{1-\rho^2_{Y_1X_2}}} \\ &= \beta_{self}\frac{1-\rho_{X_1X_2}}{\sqrt{1-\rho^2_{X_1X_2}}\sqrt{1-\rho^2_{Y_1X_2}}} \\ &= \beta_{self}\sqrt{\frac{1-\rho_{X_1X_2}}{1-\rho^2_{X_1X_2}}}\sqrt{\frac{1-\rho_{X_1X_2}}{1-\rho^2_{Y_1X_2}}}\end{aligned} \tag{3}$$

Under this scenario, $\rho_{Y_1X_2} \le \sqrt{\rho_{X_1X_2}\,\rho_{Y_1Y_2}}$, and given $0 \le \rho_{X_1X_2} \le 1$ and $0 \le \rho_{Y_1Y_2} \le 1$, so,

$$\sqrt{\frac{1-\rho_{X_1X_2}}{1-\rho^2_{X_1X_2}}} \le \sqrt{\frac{1-\rho_{X_1X_2}}{1-\rho_{X_1X_2}}} = 1 \tag{4}$$

$$\sqrt{\frac{1-\rho_{X_1X_2}}{1-\rho^2_{Y_1X_2}}} \leq \sqrt{\frac{1-\rho_{X_1X_2}}{1-\rho_{X_1X_2}\,\rho_{Y_1Y_2}}} \leq \sqrt{\frac{1-\rho_{X_1X_2}}{1-\rho_{X_1X_2}}} = 1 \quad (5)$$

So, from (3), (4) and (5), $\beta'_{self} \leq \beta_{self}$.

Similarly,

$$\beta'_{co-twin} = \rho_{X_2Y_1\,|X_1}$$

$$= \frac{\rho_{X_2Y_1} - \rho_{X_1X_2}\,\rho_{Y_1X_1}}{\sqrt{1-\rho^2_{X_1X_2}}\sqrt{1-\rho^2_{Y_1X_1}}}$$

$$= \beta_{co-twin}\frac{1-\rho_{X_1X_2}}{\sqrt{1-\rho^2_{X_1X_2}}\sqrt{1-\rho^2_{Y_1X_1}}}$$

$$= \beta_{co-twin}\sqrt{\frac{1-\rho_{X_1X_2}}{1-\rho^2_{X_1X_2}}}\sqrt{\frac{1-\rho_{X_1X_2}}{1-\rho^2_{Y_1X_1}}}$$

$$\leq \beta_{co-twin} \quad (6)$$

Under this scenario, therefore, both the conditional regression coefficients from Model 3, β'_{self} and $\beta'_{co\text{-}twin}$, attenuate with a similar magnitude, compared with the marginal regression coefficients, β_{self} from Model 1 and $\beta'_{co\text{-}twin}$ from Model 2.

2. X and Y are associated due to a causal effect of X on Y only

Under this scenario, $\rho_{X_2Y_1} = \rho_{X_2X_1}\rho_{Y_1X_1}$.

From model 2, the association between Y_1 and X_2 is conditioned on the collider Y_2, so

$$\beta_{co-twin} = \rho_{X_2Y_1|Y_2}$$

$$= \frac{\rho_{X_2Y_1} - \rho_{X_2Y_2}\rho_{Y_1Y_2}}{\sqrt{1-\rho^2_{X_2Y_2}}\sqrt{1-\rho^2_{Y_1Y_2}}}$$

$$= \frac{\rho_{X_2X_1}\rho_{X_1Y_1} - \rho_{X_2Y_2}\rho_{Y_1Y_2}}{\sqrt{1-\rho^2_{X_2Y_2}}\sqrt{1-\rho^2_{Y_1Y_2}}}$$

$$= (\rho_{X_1X_2} - \rho_{Y_1Y_2})\frac{\rho_{X_1Y_1}}{\sqrt{1-\rho^2_{X_2Y_2}}\sqrt{1-\rho^2_{Y_1Y_2}}} \quad (7)$$

So $\beta_{co\text{-}twin} > 0$ if $\rho_{X_1X_2} > \rho_{Y_1Y_2}$, and $\beta_{co\text{-}twin} < 0$ if $\rho_{X_1X_2} < \rho_{Y_1Y_2}$.

From model 3, Y_1 and X_2 is not associated given X_1 is conditioned on, i.e.,

$$\beta_{co-twin} = \rho_{X_2Y_1\,|X_1}$$

$$= \frac{\rho_{X_2Y_1} - \rho_{X_2X_1}\,\rho_{Y_1X_1}}{\sqrt{1 - \rho^2_{X_2X_1}}\sqrt{1 - \rho^2_{Y_1X_1}}}$$

$$= \frac{\rho_{X_2X_1}\,\rho_{Y_1X_1} - \rho_{X_2X_1}\rho_{Y_1X_1}}{\sqrt{1 - \rho^2_{X_2X_1}}\sqrt{1 - \rho^2_{Y_1X_1}}}$$

$$= 0 \qquad (8)$$

3. X and Y are associated due to a causal effect of Y on X only

Under this scenario, $\rho_{X_2Y_1} = \rho_{X_2Y_2}\rho_{Y_1Y_2}$.

From model 2, X_1 is a collider between Y_1 and X_2, however, it is not conditioned on. The path through Y_2 is blocked given Y_2 is conditioned on, i.e.,

$$\beta_{co-twin} = \rho_{X_2Y_1|Y_2}$$

$$= \frac{\rho_{X_2Y_1} - \rho_{X_2Y_2}\rho_{Y_1Y_2}}{\sqrt{1 - \rho^2_{X_2Y_2}}\sqrt{1 - \rho^2_{Y_1Y_2}}}$$

$$= \frac{\rho_{X_2Y_2}\rho_{Y_1Y_2} - \rho_{X_2Y_2}\rho_{Y_1Y_2}}{\sqrt{1 - \rho^2_{X_2Y_2}}\sqrt{1 - \rho^2_{Y_1Y_2}}}$$

$$= 0 \qquad (9)$$

From model 3, the association between Y_1 and X_2 is conditioned on the collider X_1, so

$$\beta'_{co-twin} = \rho_{X_2Y_1|X_1}$$

$$= \frac{\rho_{X_2Y_1} - \rho_{X_2X_1}\rho_{Y_1X_1}}{\sqrt{1 - \rho^2_{X_2X_1}}\sqrt{1 - \rho^2_{Y_1X_1}}}$$

$$= \frac{\rho_{X_2Y_2}\rho_{Y_1Y_2} - \rho_{X_2X_1}\rho_{Y_1X_1}}{\sqrt{1 - \rho^2_{X_2X_1}}\sqrt{1 - \rho^2_{Y_1X_1}}}$$

$$= (\rho_{Y_1Y_2} - \rho_{X_1X_2})\frac{\rho_{X_1Y_1}}{\sqrt{1 - \rho^2_{X_2X_1}}\sqrt{1 - \rho^2_{Y_1X_1}}} \qquad (10)$$

So $\beta'_{co\text{-}twin} < 0$ if $\rho_{X_1X_2} > \rho_{Y_1Y_2}$, and $\beta'_{co\text{-}twin} > 0$ if $\rho_{X_1X_2} < \rho_{Y_1Y_2}$.

Simulation studies of different within-pair correlations

We simulated two scenarios: 1) trait X has a causal effect on trait Y, and 2) trait Y has a causal effect on trait X, in which both traits X and Y are assumed to be continuous variables. For both scenarios, we applied ICE FALCON (fitted using Generalised Estimating Equations) using X as the predictor variable and trait Y as the outcome variable; therefore, the second scenario is reverse causation. Note that, the second scenario is equivalent to traits X and Y being reversed, i.e., trait Y as the predictor variable and trait X as the outcome variable, in the first scenario.

For the scenario in which trait X has a causal effect on trait Y, for twin i (i = 1, 2) of a monozygotic twin pair, traits X and Y were simulated as

$S_X \sim N(0, \sigma_X^2)$

$I_{Xi} \sim N(0, 1 - \sigma_X^2)$

$S_{Y\backslash X} \sim N(0, \sigma_Y^2 - b^2 * \sigma_X^2)$

$I_{Y\backslash Xi} \sim N(0, 1 - b^2 - (\sigma_Y^2 - b^2 * \sigma_X^2))$

$X_i = S_X + I_{Xi}$

$Y_i = b * X_i + S_{Y\backslash X} + I_{Y\backslash Xi}$

where

X_i: trait X for twin i

Y_i: trait Y for twin i

b: the causal effect of trait X on trait Y

S_X: the pair-shared causes influencing trait X, with a variance of σ_X^2

I_{Xi}: the individual-specific causes influencing trait X for twin i

$S_{Y/X}$: the pair-shared causes influencing trait Y that not mediated by trait X

$I_{Y\backslash Xi}$: the individual-specific causes influencing trait Y for twin i that not mediated by trait X

σ_Y^2: the variance of the total pair-shared causes influencing trait Y (including S_X and $S_{Y/X}$)

S_X, I_{Xi}, $S_{Y\backslash X}$, and $I_{Y\backslash Xi}$ follow a multivariate normal distribution with zero mean and no correlations.

Similarly, for the scenario in which Y has a causal effect on X, for twin i (i = 1, 2) of a monozygotic twin pair, traits X and Y were simulated as

$S_Y \sim N(0, \sigma_Y^2)$

$I_{Yi} \sim N(0, 1 - \sigma_Y^2)$

$S_{X\backslash Y} \sim N(0, \sigma_X^2 - b^2 * \sigma_Y^2)$

$I_{X\backslash Yi} \sim N(0, 1 - b^2 - (\sigma_X^2 - b^2 * \sigma_Y^2))$

$Y_i = S_Y + I_{Xi}$

$X_i = b * Y_i + S_{X\backslash Y} + I_{X\backslash Yi}$

where

Y_i: trait Y for twin i

X_i: trait X for twin i

b: the causal effect of trait Y on trait X

S_Y: the pair-shared causes influencing trait Y, with a variance of σ_Y^2

I_{Yi}: the individual-specific causes influencing trait Y for twin i

$S_{X/Y}$: the pair-shared causes influencing trait X that not mediated by trait Y

$I_{X\backslash Yi}$: the individual-specific causes influencing trait X for twin i that not mediated by trait Y

σ_X^2: the variance of the total pair-shared causes influencing trait X (including S_Y and $S_{X/Y}$)

S_Y, I_{Yi}, $S_{X\backslash Y}$, and $I_{X\backslash Yi}$ follow a multivariate normal distribution with zero mean and no correlations.

Under these assumptions, both traits X and Y have zero mean and unit variance, and the within-pair correlation in traits X and Y are σ_X^2 and σ_Y^2, respectively. Both traits X and Y were simulated to have nine within-pair correlations, from 0.1 to 0.9 with a step of 0.1, so a total of 81 combinations of traits X and Y were simulated. For each combination, b = 0.3, there were 10,000 twin pairs, and ICE FALCON analysis was performed.

For the scenario in which trait X has a causal effect on trait Y (Table S1), $X_{co\text{-}twin}$ was associated with Y_{self} from the Model 2, and the estimate of $\beta_{co\text{-}twin}$ was positive when $\rho_X > \rho_Y$, and negative when $\rho_X < \rho_Y$. Conditioning on X_{self} (Model 3), $X_{co\text{-}twin}$ was not associated with Y_{self}, and the estimate of $\beta_{co\text{-}twin}$ was around null, regardless of ρ_X or ρ_Y. These results are consistent with the theoretical arguments in the main text and the mathematical expressions above.

For the scenario in which trait Y has a causal effect on trait X (Table S2), $X_{co\text{-}twin}$ was not associated with Y_{self} from the Model 2, and the estimate of $\beta_{co\text{-}twin}$ was around null, regardless of ρ_X or ρ_Y. Conditioning on X_{self} (Model 3), $X_{co\text{-}twin}$ was associated with Y_{self}, and the estimate of $\beta_{co\text{-}twin}$ was negative when $\rho_X > \rho_Y$, and positive when $\rho_X < \rho_Y$. These results are consistent with the theoretical arguments in the main text and the mathematical expressions above.

From the simulation studies, ICE FALCON gave distinct patterns of regression coefficients for the two scenarios. Therefore, ICE FALCON can be used to infer X having a causal effect on Y or Y having a causal effect on X.

Simulation studies for the two examples

For the two examples, we simulated three causal scenarios each based on the observed correlational structure of the empirical data. The simulated data had the same sample size as those of the empirical data, i.e., 65 pairs for the Example 1 and 250 pairs for the Example 2. For each scenario, we conducted 1,000 simulations and applied ICE FALCON analysis.

For Example 1, the within-pair correlation in BMI was 0.79, in DNA methylation level was 0.37, the within-individual cross-trait correlation was 0.25, and the cross-twin cross-trait correlation was 0.20. We simulated the scenarios whereby BMI and DNA methylation level are associated due to: 1) familial confounding, 2) BMI having causal effect on DNA methylation level, and 3) DNA methylation level having a causal effect on BMI.

For Example 2, the within-pair correlation in baseline BMI was 0.64, in follow-up BMI was 0.60, the within-individual longitudinal correlation was 0.81, and the cross-twin cross-trait correlation was 0.63. We simulated scenarios whereby longitudinal BMI measures are associated due to: 1) familial confounding, 2) the baseline measure having a causal effect on the follow-up measure, and 3) a mixture of 1) and 2), with weights of 36% and 64%, respectively.

We compared the observed changes in regression coefficients with those expected based on the simulations, taking into account the expected variation in estimates, to determine the extent to which the simulated scenarios were consistent with the observed results. Let β_i (i = 1, 2) be the observed estimate for change in regression coefficient obtained from the ICE FALCON analysis of empirical data (i.e., change from β_{self} to β'_{self}, and change from $\beta_{co\text{-}twin}$ to $\beta'_{co\text{-}twin}$), $\bar{\theta}_i$ and $sd(\hat{\theta}_i)$ be the mean and standard deviation of the change in regression coefficient across all simulations. A test statistic (T) for goodness of fit was calculated as

$$T = \sum_{i=1}^{2} \left(\frac{\bar{\theta}_i - \beta_i}{sd(\hat{\theta}_i)} \right)^2$$

An empirical P-value was calculated based on the distribution of T across all simulations.

Table S3 and Figure S1 show the simulated results for the Example 1. No evidence was found that the scenario in which BMI has a causal effect on DNA methylation level was inconsistent with the observed results (P=0.74), while the other two scenarios were inconsistent with the observed

results (Both *P*<0.05). The ICE FALCON analysis of the empirical data of Example 1 also supports that BMI has a causal effect on DNA methylation level.

Table S4 and Figure S2 show the simulated results for the Example 2. No evidence was found that the scenario in which longitudinal BMI measures are associated due to a mixture of familial confounding and longitudinal causation was inconsistent with the observed results (*P*=0.35), while the other two scenarios were highly inconsistent with the observed results (Both *P*=0). The ICE FALCON analysis of the empirical data of the Example 2 also supports a mixture of familial confounding and longitudinal causation.

Table S1 ICE FALCON analysis results in the scenario of trait X having a causal effect on trait Y

ρ_X	ρ_Y	Coefficient	Model 1		Model 2		Model 3	
			Estimate	SE	Estimate	SE	Estimate	SE
0.1	0.1	β_{self}	0.291	0.007			0.291	0.007
		$\beta_{co\text{-}twin}$			-0.018	0.007	-0.018	0.007
0.1	0.2	β_{self}	0.293	0.007			0.291	0.007
		$\beta_{co\text{-}twin}$			-0.054	0.007	-0.018	0.007
0.1	0.3	β_{self}	0.295	0.006			0.291	0.007
		$\beta_{co\text{-}twin}$			-0.090	0.007	-0.017	0.007
0.1	0.4	β_{self}	0.296	0.006			0.290	0.007
		$\beta_{co\text{-}twin}$			-0.126	0.007	-0.017	0.007
0.1	0.5	β_{self}	0.298	0.006			0.290	0.006
		$\beta_{co\text{-}twin}$			-0.162	0.006	-0.016	0.006
0.1	0.6	β_{self}	0.299	0.005			0.291	0.006
		$\beta_{co\text{-}twin}$			-0.197	0.006	-0.015	0.006
0.1	0.7	β_{self}	0.301	0.005			0.291	0.006
		$\beta_{co\text{-}twin}$			-0.231	0.005	-0.014	0.006
0.1	0.8	β_{self}	0.302	0.003			0.291	0.006
		$\beta_{co\text{-}twin}$			-0.264	0.004	-0.012	0.006
0.1	0.9	β_{self}	0.301	0.001			0.292	0.006
		$\beta_{co\text{-}twin}$			-0.295	0.001	-0.009	0.006
0.2	0.1	β_{self}	0.289	0.007			0.291	0.007
		$\beta_{co\text{-}twin}$			0.017	0.007	-0.018	0.007
0.2	0.2	β_{self}	0.291	0.007			0.291	0.007
		$\beta_{co\text{-}twin}$			-0.019	0.007	-0.018	0.007
0.2	0.3	β_{self}	0.293	0.007			0.291	0.007
		$\beta_{co\text{-}twin}$			-0.057	0.007	-0.018	0.007
0.2	0.4	β_{self}	0.295	0.006			0.291	0.007
		$\beta_{co\text{-}twin}$			-0.095	0.007	-0.017	0.007
0.2	0.5	β_{self}	0.297	0.006			0.291	0.006
		$\beta_{co\text{-}twin}$			-0.135	0.006	-0.016	0.006
0.2	0.6	β_{self}	0.298	0.006			0.291	0.006
		$\beta_{co\text{-}twin}$			-0.175	0.006	-0.015	0.006
0.2	0.7	β_{self}	0.300	0.005			0.291	0.006
		$\beta_{co\text{-}twin}$			-0.214	0.005	-0.014	0.006
0.2	0.8	β_{self}	0.301	0.004			0.291	0.006
		$\beta_{co\text{-}twin}$			-0.253	0.004	-0.012	0.006
0.2	0.9	β_{self}	0.301	0.002			0.292	0.006
		$\beta_{co\text{-}twin}$			-0.291	0.002	-0.009	0.006
0.3	0.1	β_{self}	0.288	0.007			0.292	0.007
		$\beta_{co\text{-}twin}$			0.052	0.007	-0.018	0.007
0.3	0.2	β_{self}	0.289	0.007			0.291	0.007
		$\beta_{co\text{-}twin}$			0.017	0.007	-0.018	0.007
0.3	0.3	β_{self}	0.291	0.007			0.291	0.007
		$\beta_{co\text{-}twin}$			-0.020	0.007	-0.018	0.007

0.3	0.4	β_{self}	0.293	0.007			0.291	0.007
		$\beta_{co\text{-}twin}$			-0.060	0.007	-0.017	0.007
0.3	0.5	β_{self}	0.295	0.006			0.291	0.006
		$\beta_{co\text{-}twin}$			-0.102	0.007	-0.017	0.006
0.3	0.6	β_{self}	0.298	0.006			0.291	0.006
		$\beta_{co\text{-}twin}$			-0.147	0.006	-0.015	0.006
0.3	0.7	β_{self}	0.299	0.005			0.291	0.006
		$\beta_{co\text{-}twin}$			-0.193	0.005	-0.014	0.006
0.3	0.8	β_{self}	0.301	0.004			0.292	0.006
		$\beta_{co\text{-}twin}$			-0.239	0.004	-0.012	0.006
0.3	0.9	β_{self}	0.301	0.002			0.293	0.006
		$\beta_{co\text{-}twin}$			-0.285	0.002	-0.010	0.006
0.4	0.1	β_{self}	0.287	0.007			0.293	0.007
		$\beta_{co\text{-}twin}$			0.086	0.007	-0.018	0.007
0.4	0.2	β_{self}	0.288	0.007			0.292	0.007
		$\beta_{co\text{-}twin}$			0.054	0.007	-0.018	0.007
0.4	0.3	β_{self}	0.290	0.007			0.292	0.007
		$\beta_{co\text{-}twin}$			0.019	0.007	-0.018	0.007
0.4	0.4	β_{self}	0.292	0.007			0.292	0.007
		$\beta_{co\text{-}twin}$			-0.020	0.007	-0.017	0.007
0.4	0.5	β_{self}	0.294	0.006			0.292	0.007
		$\beta_{co\text{-}twin}$			-0.064	0.007	-0.016	0.007
0.4	0.6	β_{self}	0.296	0.006			0.292	0.006
		$\beta_{co\text{-}twin}$			-0.112	0.007	-0.015	0.006
0.4	0.7	β_{self}	0.299	0.005			0.292	0.006
		$\beta_{co\text{-}twin}$			-0.165	0.006	-0.014	0.006
0.4	0.8	β_{self}	0.301	0.004			0.292	0.006
		$\beta_{co\text{-}twin}$			-0.220	0.005	-0.012	0.006
0.4	0.9	β_{self}	0.302	0.003			0.293	0.006
		$\beta_{co\text{-}twin}$			-0.276	0.003	-0.010	0.006
0.5	0.1	β_{self}	0.286	0.007			0.294	0.008
		$\beta_{co\text{-}twin}$			0.121	0.007	-0.019	0.008
0.5	0.2	β_{self}	0.287	0.007			0.293	0.007
		$\beta_{co\text{-}twin}$			0.092	0.007	-0.018	0.007
0.5	0.3	β_{self}	0.288	0.007			0.293	0.007
		$\beta_{co\text{-}twin}$			0.060	0.007	-0.018	0.007
0.5	0.4	β_{self}	0.291	0.007			0.293	0.007
		$\beta_{co\text{-}twin}$			0.023	0.007	-0.017	0.007
0.5	0.5	β_{self}	0.293	0.007			0.293	0.007
		$\beta_{co\text{-}twin}$			-0.020	0.007	-0.016	0.007
0.5	0.6	β_{self}	0.295	0.006			0.293	0.006
		$\beta_{co\text{-}twin}$			-0.070	0.007	-0.015	0.006
0.5	0.7	β_{self}	0.298	0.006			0.293	0.006
		$\beta_{co\text{-}twin}$			-0.128	0.006	-0.014	0.006
0.5	0.8	β_{self}	0.300	0.005			0.293	0.006
		$\beta_{co\text{-}twin}$			-0.193	0.005	-0.012	0.006

0.5	0.9	β_{self}	0.302	0.003			0.293	0.006
		$\beta_{co-twin}$			-0.263	0.003	-0.010	0.006
0.6	0.1	β_{self}	0.285	0.007			0.295	0.008
		$\beta_{co-twin}$			0.154	0.007	-0.019	0.008
0.6	0.2	β_{self}	0.286	0.007			0.295	0.008
		$\beta_{co-twin}$			0.130	0.007	-0.019	0.008
0.6	0.3	β_{self}	0.287	0.007			0.294	0.008
		$\beta_{co-twin}$			0.102	0.008	-0.018	0.008
0.6	0.4	β_{self}	0.289	0.007			0.294	0.007
		$\beta_{co-twin}$			0.069	0.008	-0.017	0.007
0.6	0.5	β_{self}	0.291	0.007			0.294	0.007
		$\beta_{co-twin}$			0.030	0.008	-0.016	0.007
0.6	0.6	β_{self}	0.294	0.007			0.294	0.007
		$\beta_{co-twin}$			-0.020	0.007	-0.015	0.007
0.6	0.7	β_{self}	0.297	0.006			0.294	0.006
		$\beta_{co-twin}$			-0.081	0.007	-0.014	0.006
0.6	0.8	β_{self}	0.299	0.005			0.294	0.006
		$\beta_{co-twin}$			-0.155	0.006	-0.012	0.006
0.6	0.9	β_{self}	0.301	0.004			0.294	0.006
		$\beta_{co-twin}$			-0.243	0.004	-0.010	0.006
0.7	0.1	β_{self}	0.284	0.007			0.297	0.009
		$\beta_{co-twin}$			0.186	0.007	-0.019	0.009
0.7	0.2	β_{self}	0.285	0.007			0.296	0.009
		$\beta_{co-twin}$			0.167	0.007	-0.019	0.009
0.7	0.3	β_{self}	0.286	0.007			0.296	0.009
		$\beta_{co-twin}$			0.145	0.007	-0.019	0.009
0.7	0.4	β_{self}	0.288	0.007			0.295	0.008
		$\beta_{co-twin}$			0.118	0.008	-0.018	0.008
0.7	0.5	β_{self}	0.290	0.007			0.295	0.008
		$\beta_{co-twin}$			0.084	0.008	-0.017	0.008
0.7	0.6	β_{self}	0.292	0.007			0.295	0.007
		$\beta_{co-twin}$			0.040	0.008	-0.016	0.007
0.7	0.7	β_{self}	0.295	0.007			0.295	0.007
		$\beta_{co-twin}$			-0.019	0.007	-0.014	0.007
0.7	0.8	β_{self}	0.298	0.006			0.295	0.006
		$\beta_{co-twin}$			-0.100	0.007	-0.012	0.006
0.7	0.9	β_{self}	0.301	0.004			0.295	0.006
		$\beta_{co-twin}$			-0.209	0.005	-0.010	0.006
0.8	0.1	β_{self}	0.283	0.007			0.300	0.011
		$\beta_{co-twin}$			0.218	0.007	-0.020	0.011
0.8	0.2	β_{self}	0.284	0.007			0.299	0.011
		$\beta_{co-twin}$			0.204	0.007	-0.020	0.011
0.8	0.3	β_{self}	0.285	0.007			0.298	0.010
		$\beta_{co-twin}$			0.188	0.007	-0.019	0.010
0.8	0.4	β_{self}	0.286	0.007			0.298	0.009
		$\beta_{co-twin}$			0.168	0.008	-0.019	0.009

0.8	0.5	β_{self}	0.288	0.008			0.297	0.009
		$\beta_{co-twin}$			0.143	0.008	-0.018	0.009
0.8	0.6	β_{self}	0.290	0.008			0.297	0.008
		$\beta_{co-twin}$			0.109	0.008	-0.016	0.008
0.8	0.7	β_{self}	0.293	0.007			0.297	0.008
		$\beta_{co-twin}$			0.059	0.008	-0.015	0.007
0.8	0.8	β_{self}	0.296	0.007			0.296	0.007
		$\beta_{co-twin}$			-0.018	0.007	-0.013	0.007
0.8	0.9	β_{self}	0.300	0.005			0.296	0.006
		$\beta_{co-twin}$			-0.147	0.006	-0.010	0.006
0.9	0.1	β_{self}	0.283	0.007			0.305	0.015
		$\beta_{co-twin}$			0.249	0.007	-0.024	0.015
0.9	0.2	β_{self}	0.283	0.007			0.303	0.015
		$\beta_{co-twin}$			0.242	0.007	-0.023	0.014
0.9	0.3	β_{self}	0.284	0.007			0.303	0.014
		$\beta_{co-twin}$			0.233	0.007	-0.022	0.014
0.9	0.4	β_{self}	0.285	0.008			0.302	0.013
		$\beta_{co-twin}$			0.222	0.008	-0.021	0.013
0.9	0.5	β_{self}	0.286	0.008			0.301	0.012
		$\beta_{co-twin}$			0.207	0.008	-0.020	0.012
0.9	0.6	β_{self}	0.288	0.008			0.300	0.011
		$\beta_{co-twin}$			0.187	0.008	-0.018	0.011
0.9	0.7	β_{self}	0.291	0.008			0.300	0.010
		$\beta_{co-twin}$			0.156	0.008	-0.017	0.010
0.9	0.8	β_{self}	0.294	0.008			0.299	0.008
		$\beta_{co-twin}$			0.102	0.008	-0.014	0.008
0.9	0.9	β_{self}	0.298	0.007			0.298	0.007
		$\beta_{co-twin}$			-0.016	0.007	-0.011	0.007

Table S2 ICE FALCON analysis results in the scenario of trait Y having a causal effect on trait X

ρ_X	ρ_Y	Coefficient	Model 1		Model 2		Model 3	
			Estimate	SE	Estimate	SE	Estimate	SE
0.1	0.1	β_{self}	0.295	0.007			0.295	0.007
		$\beta_{co\text{-}twin}$			-0.022	0.007	-0.015	0.007
0.1	0.2	β_{self}	0.288	0.007			0.290	0.007
		$\beta_{co\text{-}twin}$			-0.022	0.007	0.014	0.007
0.1	0.3	β_{self}	0.277	0.007			0.285	0.007
		$\beta_{co\text{-}twin}$			-0.021	0.007	0.043	0.007
0.1	0.4	β_{self}	0.259	0.006			0.281	0.007
		$\beta_{co\text{-}twin}$			-0.019	0.007	0.073	0.007
0.1	0.5	β_{self}	0.235	0.006			0.277	0.007
		$\beta_{co\text{-}twin}$			-0.017	0.006	0.103	0.007
0.1	0.6	β_{self}	0.205	0.006			0.273	0.007
		$\beta_{co\text{-}twin}$			-0.014	0.006	0.133	0.007
0.1	0.7	β_{self}	0.166	0.005			0.269	0.007
		$\beta_{co\text{-}twin}$			-0.012	0.005	0.163	0.007
0.1	0.8	β_{self}	0.120	0.004			0.266	0.006
		$\beta_{co\text{-}twin}$			-0.009	0.004	0.194	0.006
0.1	0.9	β_{self}	0.065	0.003			0.262	0.006
		$\beta_{co\text{-}twin}$			-0.005	0.003	0.225	0.006
0.2	0.1	β_{self}	0.298	0.007			0.302	0.007
		$\beta_{co\text{-}twin}$			-0.022	0.007	-0.046	0.007
0.2	0.2	β_{self}	0.294	0.007			0.294	0.007
		$\beta_{co\text{-}twin}$			-0.022	0.007	-0.016	0.007
0.2	0.3	β_{self}	0.284	0.007			0.286	0.007
		$\beta_{co\text{-}twin}$			-0.021	0.007	0.014	0.007
0.2	0.4	β_{self}	0.269	0.006			0.278	0.007
		$\beta_{co\text{-}twin}$			-0.020	0.007	0.045	0.007
0.2	0.5	β_{self}	0.247	0.006			0.271	0.007
		$\beta_{co\text{-}twin}$			-0.018	0.007	0.076	0.007
0.2	0.6	β_{self}	0.217	0.006			0.264	0.006
		$\beta_{co\text{-}twin}$			-0.015	0.006	0.107	0.007
0.2	0.7	β_{self}	0.179	0.005			0.257	0.006
		$\beta_{co\text{-}twin}$			-0.013	0.006	0.138	0.006
0.2	0.8	β_{self}	0.131	0.005			0.250	0.006
		$\beta_{co\text{-}twin}$			-0.009	0.005	0.170	0.006
0.2	0.9	β_{self}	0.072	0.003			0.243	0.006
		$\beta_{co\text{-}twin}$			-0.006	0.003	0.202	0.006
0.3	0.1	β_{self}	0.303	0.007			0.316	0.007
		$\beta_{co\text{-}twin}$			-0.022	0.007	-0.080	0.007
0.3	0.2	β_{self}	0.301	0.007			0.304	0.007
		$\beta_{co\text{-}twin}$			-0.022	0.007	-0.049	0.007
0.3	0.3	β_{self}	0.293	0.007			0.293	0.007
		$\beta_{co\text{-}twin}$			-0.022	0.007	-0.017	0.007

0.3	0.4	β_{self}	0.280	0.007			0.282	0.007
		$\beta_{co\text{-}twin}$			-0.021	0.007	0.016	0.007
0.3	0.5	β_{self}	0.260	0.006			0.271	0.007
		$\beta_{co\text{-}twin}$			-0.019	0.007	0.048	0.007
0.3	0.6	β_{self}	0.232	0.006			0.260	0.006
		$\beta_{co\text{-}twin}$			-0.016	0.006	0.081	0.006
0.3	0.7	β_{self}	0.194	0.006			0.249	0.006
		$\beta_{co\text{-}twin}$			-0.013	0.006	0.114	0.006
0.3	0.8	β_{self}	0.144	0.005			0.239	0.006
		$\beta_{co\text{-}twin}$			-0.010	0.005	0.147	0.006
0.3	0.9	β_{self}	0.080	0.004			0.228	0.006
		$\beta_{co\text{-}twin}$			-0.006	0.004	0.181	0.006
0.4	0.1	β_{self}	0.308	0.007			0.340	0.007
		$\beta_{co\text{-}twin}$			-0.021	0.007	-0.121	0.007
0.4	0.2	β_{self}	0.308	0.007			0.324	0.007
		$\beta_{co\text{-}twin}$			-0.022	0.008	-0.087	0.007
0.4	0.3	β_{self}	0.304	0.007			0.308	0.007
		$\beta_{co\text{-}twin}$			-0.022	0.007	-0.052	0.007
0.4	0.4	β_{self}	0.293	0.007			0.293	0.007
		$\beta_{co\text{-}twin}$			-0.021	0.007	-0.017	0.007
0.4	0.5	β_{self}	0.275	0.007			0.278	0.007
		$\beta_{co\text{-}twin}$			-0.019	0.007	0.019	0.007
0.4	0.6	β_{self}	0.249	0.006			0.262	0.006
		$\beta_{co\text{-}twin}$			-0.017	0.007	0.054	0.006
0.4	0.7	β_{self}	0.211	0.006			0.247	0.006
		$\beta_{co\text{-}twin}$			-0.014	0.006	0.090	0.006
0.4	0.8	β_{self}	0.160	0.005			0.232	0.006
		$\beta_{co\text{-}twin}$			-0.011	0.005	0.126	0.006
0.4	0.9	β_{self}	0.091	0.004			0.217	0.006
		$\beta_{co\text{-}twin}$			-0.007	0.004	0.163	0.006
0.5	0.1	β_{self}	0.314	0.007			0.378	0.007
		$\beta_{co\text{-}twin}$			-0.021	0.008	-0.174	0.007
0.5	0.2	β_{self}	0.318	0.007			0.357	0.007
		$\beta_{co\text{-}twin}$			-0.022	0.008	-0.135	0.007
0.5	0.3	β_{self}	0.316	0.007			0.335	0.007
		$\beta_{co\text{-}twin}$			-0.022	0.008	-0.096	0.007
0.5	0.4	β_{self}	0.309	0.007			0.314	0.007
		$\beta_{co\text{-}twin}$			-0.021	0.008	-0.056	0.007
0.5	0.5	β_{self}	0.293	0.007			0.293	0.007
		$\beta_{co\text{-}twin}$			-0.020	0.007	-0.017	0.007
0.5	0.6	β_{self}	0.269	0.006			0.272	0.007
		$\beta_{co\text{-}twin}$			-0.018	0.007	0.023	0.007
0.5	0.7	β_{self}	0.233	0.006			0.251	0.006
		$\beta_{co\text{-}twin}$			-0.015	0.006	0.063	0.006
0.5	0.8	β_{self}	0.181	0.005			0.230	0.006
		$\beta_{co\text{-}twin}$			-0.012	0.006	0.104	0.006

0.5	0.9	β_{self}	0.106	0.004			0.209	0.006
		$\beta_{co\text{-}twin}$			-0.007	0.004	0.145	0.006
0.6	0.1	β_{self}	0.322	0.007			0.440	0.008
		$\beta_{co\text{-}twin}$			-0.020	0.008	-0.248	0.008
0.6	0.2	β_{self}	0.329	0.007			0.410	0.008
		$\beta_{co\text{-}twin}$			-0.021	0.008	-0.202	0.008
0.6	0.3	β_{self}	0.331	0.007			0.381	0.007
		$\beta_{co\text{-}twin}$			-0.021	0.008	-0.156	0.007
0.6	0.4	β_{self}	0.327	0.007			0.352	0.007
		$\beta_{co\text{-}twin}$			-0.021	0.008	-0.110	0.007
0.6	0.5	β_{self}	0.315	0.007			0.323	0.007
		$\beta_{co\text{-}twin}$			-0.020	0.008	-0.063	0.007
0.6	0.6	β_{self}	0.294	0.007			0.294	0.007
		$\beta_{co\text{-}twin}$			-0.019	0.007	-0.016	0.007
0.6	0.7	β_{self}	0.260	0.006			0.265	0.006
		$\beta_{co\text{-}twin}$			-0.016	0.007	0.031	0.006
0.6	0.8	β_{self}	0.207	0.006			0.236	0.006
		$\beta_{co\text{-}twin}$			-0.013	0.006	0.078	0.006
0.6	0.9	β_{self}	0.126	0.005			0.206	0.006
		$\beta_{co\text{-}twin}$			-0.008	0.005	0.126	0.006
0.7	0.1	β_{self}	0.331	0.007			0.547	0.008
		$\beta_{co\text{-}twin}$			-0.018	0.008	-0.365	0.008
0.7	0.2	β_{self}	0.343	0.007			0.505	0.008
		$\beta_{co\text{-}twin}$			-0.020	0.008	-0.308	0.008
0.7	0.3	β_{self}	0.350	0.007			0.462	0.008
		$\beta_{co\text{-}twin}$			-0.021	0.008	-0.250	0.008
0.7	0.4	β_{self}	0.350	0.007			0.420	0.008
		$\beta_{co\text{-}twin}$			-0.021	0.008	-0.192	0.008
0.7	0.5	β_{self}	0.343	0.007			0.378	0.007
		$\beta_{co\text{-}twin}$			-0.020	0.008	-0.133	0.007
0.7	0.6	β_{self}	0.325	0.007			0.336	0.007
		$\beta_{co\text{-}twin}$			-0.019	0.008	-0.075	0.007
0.7	0.7	β_{self}	0.294	0.007			0.294	0.007
		$\beta_{co\text{-}twin}$			-0.017	0.007	-0.016	0.007
0.7	0.8	β_{self}	0.243	0.006			0.252	0.006
		$\beta_{co\text{-}twin}$			-0.014	0.007	0.044	0.006
0.7	0.9	β_{self}	0.156	0.005			0.210	0.006
		$\beta_{co\text{-}twin}$			-0.009	0.005	0.104	0.006
0.8	0.1	β_{self}	0.343	0.008			0.766	0.009
		$\beta_{co\text{-}twin}$			-0.016	0.008	-0.594	0.009
0.8	0.2	β_{self}	0.361	0.008			0.699	0.009
		$\beta_{co\text{-}twin}$			-0.018	0.008	-0.512	0.009
0.8	0.3	β_{self}	0.374	0.008			0.632	0.009
		$\beta_{co\text{-}twin}$			-0.019	0.008	-0.430	0.009
0.8	0.4	β_{self}	0.381	0.008			0.565	0.008
		$\beta_{co\text{-}twin}$			-0.020	0.008	-0.348	0.008

0.8	0.5	β_{self}	0.380	0.008			0.498	0.008
		$\beta_{co\text{-}twin}$			-0.020	0.008	-0.265	0.008
0.8	0.6	β_{self}	0.369	0.008			0.430	0.008
		$\beta_{co\text{-}twin}$			-0.019	0.008	-0.182	0.008
0.8	0.7	β_{self}	0.343	0.007			0.363	0.007
		$\beta_{co\text{-}twin}$			-0.018	0.008	-0.099	0.007
0.8	0.8	β_{self}	0.295	0.007			0.295	0.007
		$\beta_{co\text{-}twin}$			-0.015	0.007	-0.015	0.007
0.8	0.9	β_{self}	0.204	0.006			0.227	0.006
		$\beta_{co\text{-}twin}$			-0.011	0.006	0.070	0.006
0.9	0.1	β_{self}	0.360	0.008			1.431	0.007
		$\beta_{co\text{-}twin}$			-0.012	0.008	-1.266	0.007
0.9	0.2	β_{self}	0.387	0.008			1.289	0.008
		$\beta_{co\text{-}twin}$			-0.014	0.008	-1.110	0.008
0.9	0.3	β_{self}	0.410	0.008			1.148	0.009
		$\beta_{co\text{-}twin}$			-0.015	0.008	-0.955	0.009
0.9	0.4	β_{self}	0.429	0.008			1.007	0.009
		$\beta_{co\text{-}twin}$			-0.017	0.009	-0.799	0.009
0.9	0.5	β_{self}	0.440	0.008			0.866	0.009
		$\beta_{co\text{-}twin}$			-0.017	0.009	-0.643	0.009
0.9	0.6	β_{self}	0.440	0.008			0.724	0.009
		$\beta_{co\text{-}twin}$			-0.018	0.009	-0.487	0.009
0.9	0.7	β_{self}	0.425	0.008			0.582	0.009
		$\beta_{co\text{-}twin}$			-0.018	0.009	-0.330	0.009
0.9	0.8	β_{self}	0.386	0.008			0.440	0.008
		$\beta_{co\text{-}twin}$			-0.017	0.008	-0.172	0.008
0.9	0.9	β_{self}	0.296	0.007			0.296	0.007
		$\beta_{co\text{-}twin}$			-0.013	0.007	-0.014	0.007

Table S3 Simulation results for Example 1

Causal scenario	Regression coefficient	Observed estimate	Mean	T	P
Familial confounding	β_{self} in Model 1	0.132	0.117	2.21	0.022
	$\beta_{co\text{-}twin}$ in Model 2	0.086	0.119		
	β'_{self} in Model 3	0.131	0.068		
	$\beta'_{co\text{-}twin}$ in Model 3	0.003	0.077		
	Change from β_{self} to β'_{self}	-1.38	-48.94		
	Change from $\beta_{co\text{-}twin}$ to $\beta'_{co\text{-}twin}$	-96.60	-41.50		
BMI having a causal effect on methylation level	β_{self} in Model 1	0.132	0.130	0.003	0.737
	$\beta_{co\text{-}twin}$ in Model 2	0.086	0.079		
	β'_{self} in Model 3	0.131	0.131		
	$\beta'_{co\text{-}twin}$ in Model 3	0.003	-0.002		
	Change from β_{self} to β'_{self}	-1.38	0.77		
	Change from $\beta_{co\text{-}twin}$ to $\beta'_{co\text{-}twin}$	-96.60	-95.62		
Methylation level having a causal effect on BMI	β_{self} in Model 1	0.132	0.166	2.13	0.056
	$\beta_{co\text{-}twin}$ in Model 2	0.086	0.000		
	β'_{self} in Model 3	0.131	0.249		
	$\beta'_{co\text{-}twin}$ in Model 3	0.003	-0.147		
	Change from β_{self} to β'_{self}	-1.38	56.62		
	Change from $\beta_{co\text{-}twin}$ to $\beta'_{co\text{-}twin}$	-96.60	-76.46		

Table S4 Simulation results for Example 2

Causal scenario	Regression coefficient	Observed estimate	Mean	χ^2	P
Familial confounding	β_{self} in Model 1	0.810	0.628	62.92	0
	$\beta_{co\text{-}twin}$ in Model 2	0.732	0.612		
	β'_{self} in Model 3	0.731	0.426		
	$\beta'_{co\text{-}twin}$ in Model 3	0.147	0.367		
	Change from β_{self} to β'_{self}	-9.72	-32.10		
	Change from $\beta_{co\text{-}twin}$ to $\beta'_{co\text{-}twin}$	-76.23	-39.95		
Baseline BMI measure having a causal effect on follow-up BMI measure	β_{self} in Model 1	0.810	0.831	267.27	0
	$\beta_{co\text{-}twin}$ in Model 2	0.732	0.609		
	β'_{self} in Model 3	0.731	0.831		
	$\beta'_{co\text{-}twin}$ in Model 3	0.147	-0.0004		
	Change from β_{self} to β'_{self}	-9.72	0.01		
	Change from $\beta_{co\text{-}twin}$ to $\beta'_{co\text{-}twin}$	-76.23	-100.22		
A mixture of familial confounding and longitudinal causation	β_{self} in Model 1	0.810	0.824	1.72	0.351
	$\beta_{co\text{-}twin}$ in Model 2	0.732	0.770		
	β'_{self} in Model 3	0.731	0.714		
	$\beta'_{co\text{-}twin}$ in Model 3	0.147	0.181		
	Change from β_{self} to β'_{self}	-9.72	-13.37		
	Change from $\beta_{co\text{-}twin}$ to $\beta'_{co\text{-}twin}$	-76.23	-76.51		

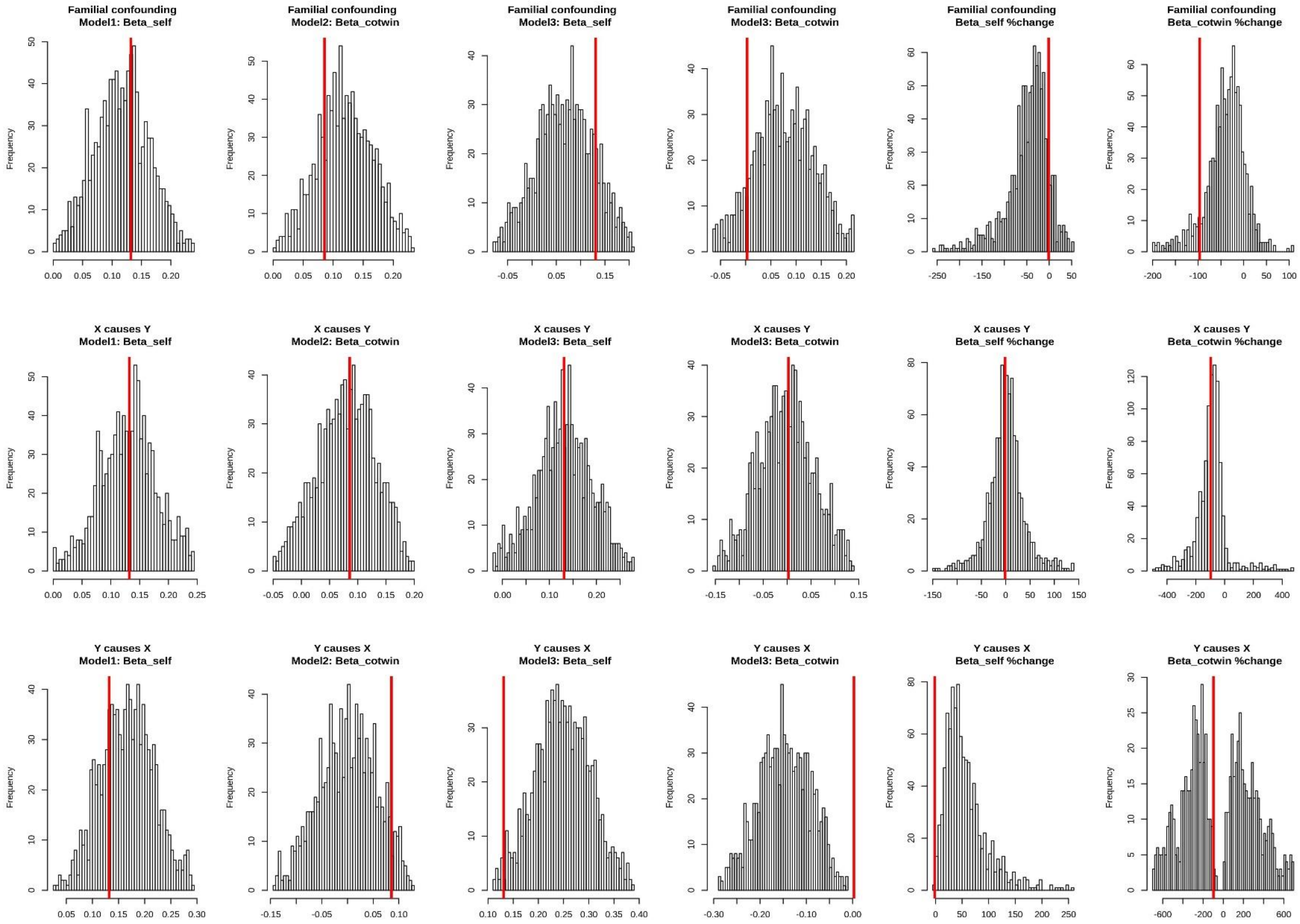

Figure S1 Distribution of the parameter estimate from the simulation study for the Example 1
The red vertical line is the observed parameter estimate obtained from the ICE FALCON analysis of empirical data

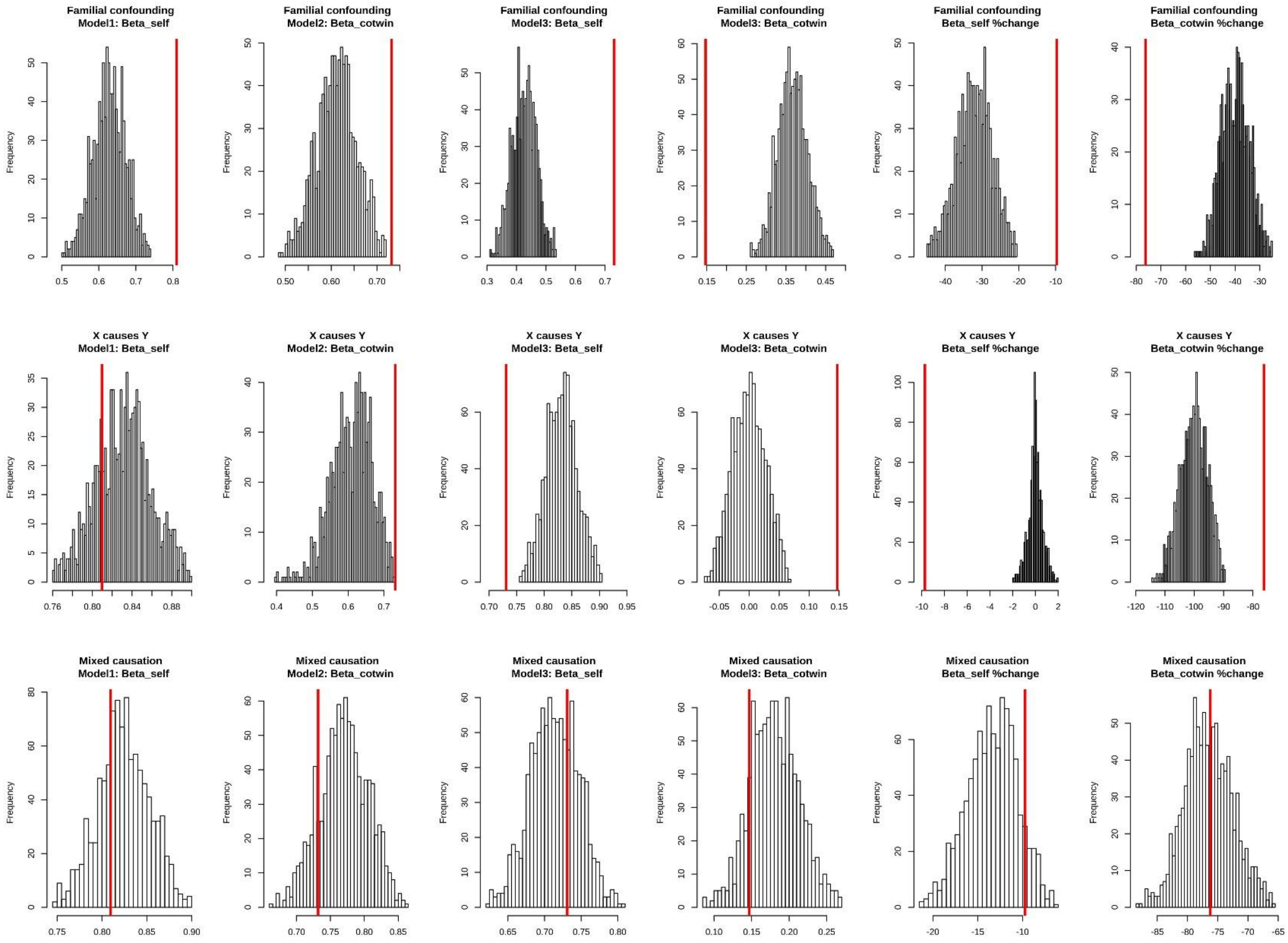

Figure S2 Distribution of the parameter estimate from the simulation study for the Example 2
The red vertical line is the observed parameter estimate obtained from the ICE FALCON analysis of empirical data
